# One Year Evaluation of Material Properties Changes of Polylactide Parts in Various Hydrolytic Degradation Conditions

**DOI:** 10.3390/polym11091496

**Published:** 2019-09-13

**Authors:** Angela Andrzejewska

**Affiliations:** Department of Biomedical Engineering, Faculty of Mechanical Engineering, UTP University of Science and Technology in Bydgoszcz, Prof. S. Kaliskiego 7 Avenue, 85-796 Bydgoszcz, Poland; angela.andrzejewska@utp.edu.pl

**Keywords:** polylactide, hydrolytic degradation, mechanical properties

## Abstract

Biodegradable biocompatible materials are widely used in medical applications. Determining the possibility of using biodegradable materials depends on determining the changes in their parameters over time due to degradation. The current scientific research on biodegradable materials has presented results based on research methods characterized by the different geometry and cross-section size of the specimen, type of degradation medium, or different pH value of the medium or maximum degradation time. This paper presents the results of a one-year study on the influence of the type of degradation medium on the changes in mechanical behavior and the uptake of the degradation medium by biodegradable specimens with large cross-sections. In addition, a prototype of a test stand was created, which allowed for the specimens to be stored vertically to ensure regular medium exposure and eliminate the interaction of the surface of the tested specimens with the sides of the container. The obtained results allowed the statistical significance of differences in the mechanical parameters determined in the uniaxial tensile test after 2, 4, 6, 12, 26, 39, and 52 weeks of degradation to be indicated depending on the type of degradation medium. It was proven that the changes in mechanical behavior depend on the percentage change in the mass of the specimens during degradation. The percentage change in mass depends on the type of degradation medium. Based on the results of this research, it was noted that in long-term degradation above 12 weeks, buffered sodium chloride solution is the optimal choice for the degradation medium. However, distilled water or physiological saline solution can be used as an alternative during the degradation period for up to 12 weeks.

## 1. Introduction

Polymeric materials, just like metals and ceramics, are widely used in medical applications. In the group of polymeric materials, synthetic, biological, and hybrid materials can be distinguished. In [1], the authors presented a detailed list of synthetic polymeric materials for biomedical applications. These materials are given in detail: polyolefins, poly(tetrafluoroethylene) (PTFE), poly(vinyl chloride) (PVC), silicone, methacrylates, polyesters, polyethers, polyamides, and polyurethanes. In addition, the materials were divided and described according to their intended use to produce temporary in vivo applications; general surgical implants; orthopedic implants; vascular and cardio-vascular intervention; plastic, reconstructive, and cosmetic surgery as well as ophthalmology, dentistry, or neurosurgery. 

Synthetic polymers can also be classified according to their biodegradability under physiological conditions. The group of synthetic, biodegradable polymers deserves special attention. After fulfilling their function, they are cleared out of the body by natural methods. Synthetic, biodegradable polymers are used to produce surgical sutures [2,3], bone anastomosis plates [4], materials for tissue scaffolds [5,6,7], and scaffolds for repairing tendons and ligaments [8].

Due to the increasing interest and wide variety of application possibilities of biodegradable, biocompatible polymers, it is necessary to evaluate the changes in the behavior of these materials under the influence of the degradation of the aqueous conditions. The test procedure for biodegradable materials, especially for surgical applications, has been defined in ISO 15814, which describes in detail the type of degradation medium used, degradation times, and test methods. The standard recommends the use of buffered sodium chloride solution, pH = 7.4, heated to 37 °C. The pH of the medium should be controlled. If necessary, the medium should be buffered or replaced periodically. The standard also specifies the degradation intervals between the verification of selected parameters. The intervals of 0, 2, 4, 8, 16, and 26 weeks are recommended for short-term degradable polymers (e.g., for PLGA), and for long-term degradable polymers (e.g., pure PLA) of 0, 6, 12, 26, 39, and 52 weeks, respectively. 

In the research described in scientific publications, authors have used different specimen types, types of degradation medium, and maximum degradation times as well as the intervals between the individual intervals of the verification of changes within the material. The adopted test methods apply to biocompatible materials such as polylactide and its enantiomers (PLLA, PDLLA) [9,10,11], polyglycolide [10], polycaprolactone [11,12], polylactide-co-glycolide [13,14], polyurethanes [15], trimethylene polycarbonate [16], polylactide with polycaprolactone [17], polydoxanone [17], and polylactide based copolymers, for example, polylactide-co-glycolide composites with calcium dihydrogen phosphate, calcium hydrophosphate, calcium phosphate and calcium carbonate [18], polylactide-co-δ-valerolactone [19], polylactide with polycaprolactone [17,20], and the poly-DL-lactide-co-polycarbonate of trimethylene [16]. Composites with biodegradable materials (e.g., polylactide with carbon fiber [21]) are also subjected to degradation tests.

In the study by Barbeck et al. [9], specimens were placed in a simulated physiological fluid (SBF) heated to 37 °C and stored for eight weeks. In the paper by Bartkowiak-Jowsa et al. [10], the process of hydrolytic degradation was carried out in distilled water at a pH 7.0 and temperature 37 °C. The total time of the storage of specimens in the medium was 24 months, and the studies were performed at monthly intervals in the first year and at bimonthly intervals in the second year. In the studies Scaffaro et al. presented in [11], specimens were stored in a buffer solution with a variable pH of 4.0, 7.0, and 10.0 and heated to 37 °C. The incubation time was 50 days and the tests were performed after 4, 21, and 50 days. The authors of subsequent papers [12,13,14,15,16,18,20] used buffered sodium chloride (PBS) at pH = 7.4, which was heated to 37 °C, as a degradation medium. These studies were characterized by a variable incubation period of 90 days [12], 12 months [13], 30 days [14], 38 weeks [15], 53 weeks [16], and 8 weeks [18,20]. 

In the study by Vieira et al. [17], three different degradation media (i.e., water, PBS, and sodium chloride (NaCl) solution) were investigated and heated to 37 °C. The total time of specimen degradation was 12 or 28 weeks. In the case of the studies Fernández et al. presented in [19], the experiments were also conducted in PBS, but in contrast to the previously quoted studies, the pH of the solution was 7.2. The incubation time of the specimens was 98 days. However, in Liu et al. [21], the degradation medium was the HANKs solution, which, as above, was heated to 37 °C. The total conditioning time was 49 or 73 weeks.

Summarizing the above analyses of the current state of knowledge, the diversity of the adopted research methodology for determining the influence of degradation on selected parameters of tested biodegradable materials can be observed. Moreover, most of the tests were conducted for specimens with small cross-sections (e.g., surgical sutures). 

The aim of this study was to determine the influence of the most often used types of degradation media on the rate of mechanical behavior changes and the absorption of the degradation medium by a biocompatible polymeric material with a long degradation time. The results of this research will be used to determine the limits of applicability of selected media in the degradation of the model of biodegradable material.

## 2. Materials and Methods 

The aim of the work was achieved by using appropriate materials and research methods. In order to verify the influence of the medium type on the changes in the behavior of the polymeric material as a model material, granules made of biodegradable polylactide with the commercial name of Ingeo™ Biopolymer 3100HP (NatureWorks LLC, Minnetonka, MN, USA) were used. The material was characterized by a density of 1.24 g/cm^3^ and a melt mass flow rate (MFR) of 24 g/10 min was selected. Specimens from biodegradable material were formed by injection molding on a hybrid Engel E-Victory 310/110 (Engel Austria GmbH, Schwertberg, Austria) injection molding machine equipped with an Engel Viper 6 robot. The geometry of the molds used in the test was in accordance with ISO 527-1, type 1A.

The polymer specimens were exposed to hydrolytic degradation in three degradation media (i.e., buffered saline solution – PBS (Biocorp Polska Sp. z o.o, Warsaw, Poland), 0.9% saline solution (0.9% NaCl, self-made), and distilled water (H_2_O_dest._, self-made)). The degradation time was 0, 2, 4, 6, 12, 26, 39, and 52 weeks. During the degradation phase, the specimens were stored vertically in relation to their longer axis in a prepared in-house research stand. The test stand was registered for patent protection in the Patent Office of the Republic of Poland under number (W.127335, 15.05.2018). The prototype of the stand allowed for a constant temperature of 37 ± 1 °C to be maintained. The pH value of the degradation medium was controlled using an Elmetron CP-411 pH-meter (ELMETRON, Zabrze, Poland). The medium was changed at least every four weeks.

Tests of the mechanical properties were performed on an INSTRON ElectroPuls E3000 (INSTRON, Norwood, MA, USA) equipped with an electromagnetic actuator with a force range of ±3 kN. The speed of moving the crosshead of the testing machine was 1 mm/min. The INSTRON static clip-on extensometer was used to determine the material deformation and modulus of elasticity. The distance between the measuring arms was 12.5 mm. Based on the measured values of stresses and deformations, the following values were determined: tensile strength (*σ*_M_), breaking strength (*σ*_B_), Young modulus (*E*), and toughness (*Q*). In each group, n = 8 specimens were tested. Specimens were tested at room temperature (*T* = 23 ± 2 °C, humidity 50 ± 10%) after removal from the medium and dried with a paper towel.

The change in the relative mass of the specimens during degradation (Δ*m*) was determined from the data collected during the weighing process. The initial mass (*m*_0_) of the specimen was determined before it was placed in the degradation medium and after a specified time of degradation, the mass was measured in time (*m*_t_). The mass change was estimated based on the following equation: Δ*m = (m*_t_ *− m*_0_*)/m*_0_ × 100%. AS 220.X2, RADWAG (Radom, Poland) laboratory scales were used to determine the mass of specimens.

The obtained results were analyzed using software such as GraphPad and Excel, and descriptive statistics and statistical significance tests were used. The normality of the distribution of quantitative variables was determined using the Shapiro–Wilk test. Verification was carried out at the level of significance of α = 0.05. The statistical significance of the differences in the achieved results was determined with the use of one-way ANOVA variance analysis. The aim of these analyses was to verify the hypothesis of the equality of mean values of the tested variable in several populations (*k ≥ 2*). Fisher′s LSD test was used to determine the differences of the studied results.

## 3. Results and Discussion

**Figure 1:**
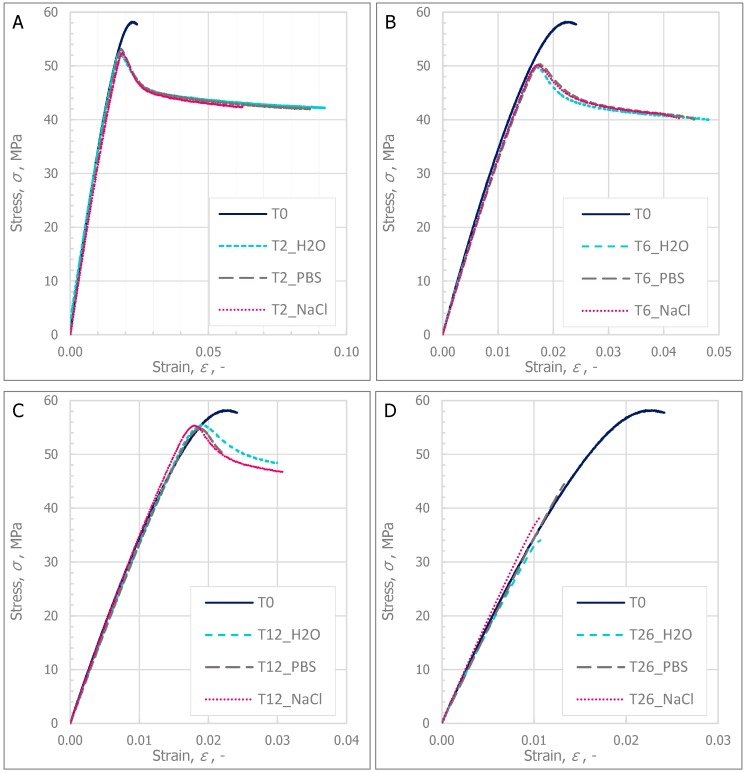
The stress–strain curves of the specimens during degradation in various mediums: (**A**) Comparison between specimens before degradation (T0) and after two weeks of degradation; (**B**) Comparison between specimens before degradation and after six weeks of degradation; (**C**) Comparison between specimens before degradation and after twelve weeks of degradation; (**D**) Comparison between specimens before degradation and after twenty-six weeks of degradation.

**Figure 2:**
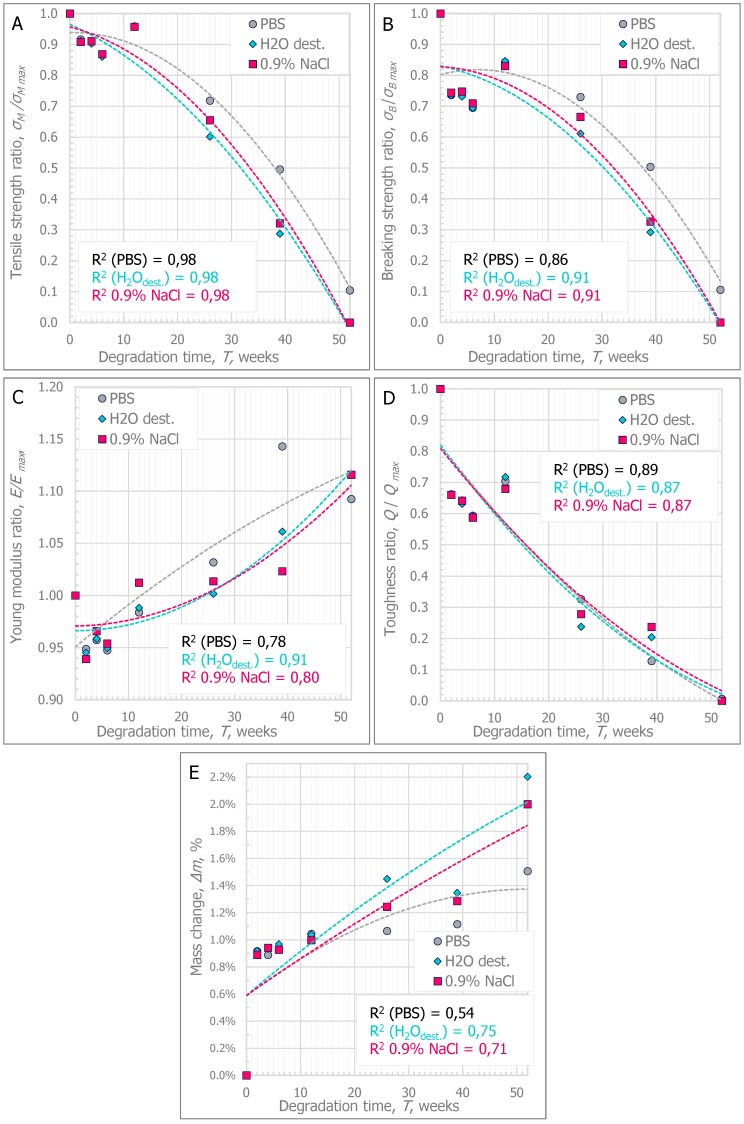
Tendencies of the changes in material parameters during degradation: (**A**) Tensile strength ratio; (**B**) Breaking strength ratio; (**C**) Young modulus ratio; (**D**) Toughness ratio; (**E**) Mass change.

**Figure 3:**
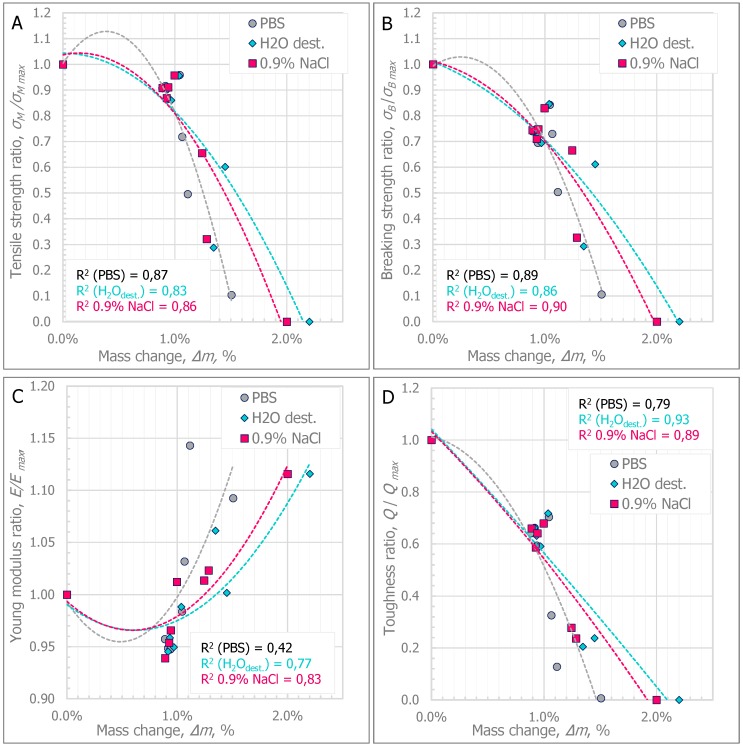
Tendencies of changes in the material parameters in relation to mass change: (**A**) Tensile strength ratio; (**B**) Breaking strength ratio; (**C**) Young modulus ratio; (**D**) Toughness ratio.

Four parameters (tensile strength (*σ*_M_), breaking strength (*σ*_B_), Young modulus (*E*), and absorbed energy (*Q*)) were used in the analysis of the influence of the type of degradation medium on the changes in the mechanical behavior of the material. Changes in the mentioned mechanical parameters obtained from the uniaxial tensile test were observed with relation to the time of degradation (in weeks) and to the mass change (Δ*m*). Figure 1 shows an example of the stress–strain curves of the specimens during degradation at selected intervals in various mediums in relation to the unconditioned specimens. However, the tendencies of changes are presented in Figure 2 and Figure 3.

The stress–strain curves presented in Figure 1 show that in the case where the specimens were immersed in solution, the relative elongation of the specimens increased and the tensile strength decreased. Due to the degradation processes over six months of immersion time, the stress–strain curves changed. In this case, the relative elongation does not increase with the decrease in tensile strength, and the specimens cracked when the maximum tensile force was reached.

The tendencies of changes in the mechanical parameters were described with a model of square function. Comparisons of the statistical significance of the differences in the results were made in two ways. In the first analysis, the differences between the values obtained in each week of degradation for each type of medium were compared. In the second stage, the significance of the differences between three types of degradation media in specific weeks of degradation were compared.

The value of the coefficients of determination R^2^ shows that the method applied to the description of degradation tendency with a square function allows for a value of the regression line fitting above R^2^ = 0.8 to be obtained, thus, giving a good curve fitting for most of the analyzed parameters (i.e., the comparison of the relationship between the change in tensile strength, braking strength, and toughness during degradation). The adopted model of the Young modulus changed and the relative mass changed dependent on the time of degradation by means of a square function showed on average fit.

In Figure 2A, the average tendencies of decrease in tensile strength during degradation depending on the type of degradation medium, are shown. Table 1 shows the mean values with the standard deviation and the median of the tensile strength parameter. In the case of specimens degraded in 0.9% sodium chloride solution and distilled water, the tensile strength in T52 was not determined because the specimens broke in the grips of the testing machine. In the table below, the “*x*” symbol is used. This statement also refers to the other parameters. Additionally, it was assumed that the specimens marked with the symbol *“x”* reached values close to 0 MPa (*σ*_M_, *σ*_B_), 0 MJ/m^3^ (*Q*), and ≥4000 MPa (*E*; according to the PBS test). 

Based on the presented curves, it was observed that the specimens stored in PBS showed a slower tendency toward a decrease in tensile strength than the specimens stored in 0.9% sodium chloride solution and distilled water. The rate of degradation in this case was stated as a 50% decrease in tensile strength in relation to the value determined at T0. Specimens stored in PBS peaked at T39. In contrast to the specimens stored in PBS, the specimens stored in 0.9% NaCl or H_2_O_dest._ were characterized by a decrease in tensile strength of almost 70%. In order to compare the differences between the strength values determined in different weeks of degradation depending on the type of medium used, they were significantly different for each analyzed medium. Comparisons between three media types in a given week of degradation demonstrated that in the weeks between T2 and T12, the medium type did not significantly affect the differences between the values obtained for PBS, 0.9% NaCl, and H_2_O_dest._. Only in the period from T26 to T52 did the type of medium influence the strength values reached by the specimens for the specimens degraded in PBS, which confirmed the observed tendency of a slower decrease in the strength values than in the other two media. No statistically significant differences were found in the period from T26 to T52 for the comparison of 0.9% NaCl and H_2_O_dest._.

In Figure 2B, the average tendencies of the decrease in breaking strength during the degradation depending on the type of the applied degradation medium are presented. Table 2 shows the mean values with their standard deviation and the median of the breaking strength parameter.

It was observed that in the period from T26 to T52, the value of the breaking strength of the specimens was equal to their tensile strength value. Similarly, as in the case of the tensile strength parameter, the statistical significance of the differences in breaking strength between the weeks of degradation in each of the three media and between the three media in a selected week of degradation is shown.

In Figure 2C, the average growth tendencies of the Young modulus during degradation depending on the type of the medium are shown. Table 3 shows the mean values with their standard deviation and the median value of the Young modulus parameter. 

At the beginning of T2-to-T6 degradation, a decrease in the Young modulus by about 5% was observed. After T12, this value slightly increased by 5–15% relative to T0. However, no statistically significant differences were found between the influence of the degradation medium on the changes in the Young modulus. This trend was confirmed throughout the degradation period and in comparisons between the media in a given week of degradation.

In Figure 2D, the average tendencies of the toughness decrease during the degradation depending on the type of the medium are presented. Table 4 presents the mean values with their standard deviation and the median of the toughness parameter.

Based on the determined tendencies of decrease in the toughness parameter, it can be observed that in the period T2-to-T12, its value decreased by nearly 40%, and in the following weeks of degradation, it decreased to about 80–90% of the initial value. For T52 specimens, the toughness parameter, which was close to 0 MJ/m^3^, was determined only for PBS specimens. 

In Figure 1E, the average tendencies of increased percentage change of mass during degradation depending on the type of the chosen medium are presented. Table 5 presents the values of mean, standard deviation, and the median of the percentage mass change parameter.

On the grounds of the determined tendencies of increase of the percentage parameter of mass change, it can be observed that in the period from T2 to T12, its value increased by nearly 1%, and in subsequent weeks of degradation, it increased to over 2% of the initial value. In the case of the specimens tested in T52, the specimens conditioned in distilled water and 0.9% saline solution achieved a mass increase of more than 2%, while in the case of the specimens stored in PBS, the value did not exceed 1.5% of the initial value.

The analyses proved the statistical significance of the differences in toughness between the specimens studied in the three media during the degradation period. Moreover, from T12, the type of degradation medium had an influence on the results achieved. Specimens stored in PBS needed more energy to destroy them than specimens stored in 0.9% NaCl or H_2_O_dest._. No statistically significant differences between the use of 0.9% NaCl or H_2_O_dest._ were found in studies over 12 weeks.

In this study, the mechanical tests showed a higher value of strength and toughness at 12 weeks than the specimens at a short degradation time. A similar phenomenon was described in Tokiwa et al. [22] where the authors indicated that the phenomenon of crystallization during degradation was the justification for the changes affecting the improvement of material strength. As a result of crystallization, new bonds developing in the polymer chain reduce their susceptibility to creep [10].

Figure 3A–D show the curves of the average tendencies of changes in specific mechanical parameters in relation to the percentage change in the mass of specimens. 

Initially, the change in the mass of the specimens is related to the absorption of the medium by the polymer specimen. The percentage change in mass is then stabilized. This phenomenon has been observed by other researchers in relation to polymer and elastomer tests, which is described in the paper by Younes et al. [23]. During the stabilization period, the relative mass change value is between approximately 0.89% and approximately 0.95% of the initial mass. In addition, it has been pointed out that during this period, changes in mechanical parameters are followed by no more than a 30% decrease in the tensile strength, breaking strength, or Young modulus, and a nearly 50% decrease in toughness. This period occurs approximately to the twelfth week of degradation, where based on the statistical analyses, there were no significant differences in the type of degradation medium used.

A progressive increase in the percentage value of mass change was found over 12 weeks of degradation and the mass of the specimens increased above 1% relative to the initial mass. Increasing the percentage change in the specimen mass above 1% is associated with a change in material properties. In each analyzed case, the cracking method of the specimen changed from ductile failure to brittle failure (σ*_M_ =* σ*_B_*). In addition, the increase in medium absorption, and thus, the percentage change in specimen mass, of more than 1.5%, significantly hindered the test process. The specimens stored in H_2_O_dest_. and 0.9% NaCl during the T52 week were characterized by a mass change of more than 2%, which made it impossible to test them. In both cases, the test specimens broke in the grips of the testing machine.

## 4. Summary and Conclusions

The results of the research presented in this paper compared the influence of the type of medium on the speed of changes in mechanical parameters during 52 weeks of degradation as well as the absorption level of the medium as defined by the percentage change in the mass of the specimens. The obtained results showed, in general, a lower strength of the specimens stored in 0.9% NaCl and H_2_O_dest._. The phenomenon of a faster decrease of strength parameters was observed for these specimens from T26. Moreover, based on statistical analyses, it was shown that if the degradation is carried out for no longer than 12 weeks, the type of medium does not significantly influence the changes of the strength parameters. In the time frame of up to 12 weeks, no significantly higher degradation medium uptake by the tested specimens was found. Only after 12 weeks was it observed that specimens conditioned in 0.9% NaCl and H_2_O_dest._ had significantly higher medium consumption than specimens stored in PBS.

The presented model of changes in material properties due to medium absorption shows how the different chemical composition of the medium can significantly influence the rate of polymer material degradation. Therefore, a quantitative approach to degradation changes should lead to a more rational choice of degradation medium in future research.

The observed tendencies of changes in strength parameters allow for the optimal medium to be chosen (i.e., buffered saline solution), which, due to its properties, opposes significant changes in pH due to the hydrolysis of a biocompatible biodegradable material. Moreover, linking the properties of the buffer solution with the properties of physiological fluids in a living human or animal body allowed us to conclude that it corresponded much better to the internal environment than to distilled water or a 0.9% sodium saline solution. Therefore, a slower degradation rate in PBS than in other degradation media is a phenomenon of high demand.

The results presented in the paper contribute to the current state of knowledge by analyzing the influence of the type of degradation medium on changes in the mechanical behavior of biodegradable biocompatible materials for specimens with larger cross-sections than in the case of surgical sutures or plates for small bone anastomoses. Nevertheless, against the background of the obtained results, the geometric dimensions of biodegradable biomedical solutions used in bone anastomoses should be studied in more detailed studies.

The authors in [24] showed that the surface of a matrix made of polylactide was less susceptible to the rate of hydrolysis than its internal part and noted that if the polymeric matrix was initially crystalline or crystallizes during degradation, the internal part of a large-sized product would degrade faster than its surface without the formation of hollow residual structures.

Moreover, due to the noted higher uptake of the degradation medium by specimens stored in 0.9% NaCl and H_2_O_dest._, future degradation tests should be extended with an analysis of the composition of the medium after degradation. In the studies described in the paper, only the changes in the pH of the medium were verified.

## 5. Patents

It was possible to maintain the conditions of hydrolytic degradation by developing a prototype of a test stand. The improved design of the test stand was submitted for protection to the Patent Office of the Republic of Poland under no. W.127335 (15.05.2018).

## Figures and Tables

**Table 1:** Mean and median values of the tensile strength parameter (*σ_M_*, MPa) during degradation.

Value of *σ_M_*	Medium 1:PBS	Medium 1:PBS	Medium 2:0.9% NaCl	Medium 2:0.9% NaCl	Medium 3:H_2_O_dest._	Medium 3:H_2_O_dest._
Time, T, Weeks	Mean ± STD	Median	Mean ± STD	Median	Mean ± STD	Median
0	57.92 ± 0.66	58.22	57.92 ± 0.66	58.22	57.92 ± 0.66	58.22
2	53.02 ± 0.13	53.01	52.62 ± 0.48	52.39	52.78 ± 0.34	52.65
4	52.62 ± 0.22	52.47	52.76 ± 0.20	52.81	52.28 ± 0.29	52.18
6	50.08 ± 0.31	50.25	50.33 ± 0.22	50.23	49.83 ± 0.48	49.91
12	55.68 ± 0.42	55.71	55.44 ± 0.26	55.39	55.37 ± 0.30	55.50
26	42.12 ± 1.88	41.43	37.94 ± 3.38	38.74	34.86 ± 1.28	34.80
39	29.59 ± 3.26	27.73	18.62 ± 3.20	17.39	16.67 ± 1.95	16.65
52	6.03 ± 2.47	5.09	x	x	x	x

**Table 2:** Mean and median values of the breaking strength parameter (*σ_B_*, MPa) during degradation.

Value of *σ_B_*	Medium 1:PBS	Medium 1:PBS	Medium 2:0.9% NaCl	Medium 2:0.9% NaCl	Medium 3:H_2_O_dest._	Medium 3:H_2_O_dest._
Time, T, Weeks	Mean ± STD	Median	Mean ± STD	Median	Mean ± STD	Median
0	57.00 ± 0.42	56.96	57.00 ± 0.42	56.96	57.00 ± 0.42	56.96
2	41.98 ± 0.33	41.92	42.42 ± 0.99	42.26	41.92 ± 0.41	42.14
4	42.20 ± 0.43	42.01	42.61 ± 0.32	42.54	41.68 ± 0.26	41.73
6	39.61 ± 0.44	39.44	40.47 ± 0.87	40.30	39.58 ± 0.34	39.58
12	48.04 ± 1.39	47.84	47.30 ± 1.24	46.64	48.22 ± 0.71	47.91
26	41.61 ± 1.88	41.43	37.94 ± 3.38	38.74	34.86 ± 1.28	34.80
39	28.72 ± 3.26	27.73	18.62 ± 3.20	17.39	16.67 ± 1.95	16.65
52	6.03 ± 2.47	5.09	x	x	x	x

**Table 3:** Mean and median values of the Young modulus parameter (*E*, MPa) during degradation.

Value of *E*	Medium 1:PBS	Medium 1:PBS	Medium 2:0.9% NaCl	Medium 2:0.9% NaCl	Medium 3:H_2_O_dest._	Medium 3:H_2_O_dest._
Time, T, Weeks	Mean ± STD	Median	Mean ± STD	Median	Mean ± STD	Median
0	3585.45 ± 123.32	3598.36	3585.45 ± 123.32	3598.36	3585.45 ± 123.32	3598.36
2	3419.68 ± 60.50	3382.19	3366,91 ± 49.81	3368.43	3389.44 ± 92.53	3382.61
4	3426.34 ± 27.08	3432.22	3462.73 ± 98.92	3462.27	3436.71 ± 54.45	3436.74
6	3399.84 ± 29.20	3386.44	3419.93 ± 72.91	3401.63	3404.59 ± 26.06	3410.28
12	3540.73 ± 53.45	3517.70	3629.45 ± 54.69	3662.88	3543.33 ± 39.08	3545.98
26	3703.38 ± 291.30	3599.78	3634.50 ± 139.96	3582.97	3591.46 ± 116.25	3635.94
39	3972.72 ± 296.05	3999.60	3668.81 ± 170.59	3701.04	3804.92 ± 350.35	3690.25
52	3916.82 ± 829.14	3914.08	x	x	x	x

**Table 4:** Mean and median values of the toughness parameter (*Q,* MJ/m^3^) during degradation.

Value of *Q*	Medium 1: PBS	Medium 1: PBS	Medium 2: 0.9% NaCl	Medium 2: 0.9% NaCl	Medium 3: H_2_O _dest._	Medium 3: H_2_O _dest._
Time, T, weeks	Mean ± STD	Median	Mean ± STD	Median	Mean ± STD	Median
0	0.812 ± 0.032	0.821	0.812 ± 0.032	0.821	0.812 ± 0.032	0.821
2	0.538 ± 0.005	0.536	0.536 ± 0.007	0.538	0.538 ± 0.017	0.536
4	0.522 ± 0.008	0.525	0.521 ± 0.007	0.522	0.513 ± 0.012	0.508
6	0.482 ± 0.013	0.487	0.477 ± 0.003	0.476	0.480 ± 0.004	0.479
12	0.571 ± 0.004	0.570	0.552 ± 0.005	0.553	0.583 ± 0.006	0.584
26	0.265 ± 0.033	0.276	0.226 ± 0.038	0.236	0.193 ± 0.015	0.192
39	0.104 ± 0.032	0.096	0.050 ± 0.014	0.045	0.038 ± 0.006	0.040
52	0.005 ± 0.004	0.004	x	x	x	x

**Table 5:** Mean and median values of the mass change parameter (Δ*m,* %) during degradation.

Value of Δ*m*	Medium 1: PBS	Medium 1: PBS	Medium 2: 0.9% NaCl	Medium 2: 0.9% NaCl	Medium 3: H_2_O_dest._	Medium 3: H_2_O_dest._
Time, T, Weeks	Mean ± STD	Median	Mean ± STD	Median	Mean ± STD	Median
0	x	x	x	x	x	x
2	0.90 ± 0.03	0.91	0.91 ± 0.03	0.91	0.90 ± 0.04	0.92
4	0.91 ± 0.04	0.92	0.96 ± 0.08	0.98	0.95 ± 0.02	0.96
6	0.95 ± 0.01	0.95	0.89 ± 0.13	0.95	0.92 ± 0.07	0.95
12	1.04 ± 0.03	1.03	1.04 ± 0.02	1.05	1.00 ± 0.03	1.01
26	1.07 ± 0.01	1.07	1.45 ± 0.33	1.25	1.24 ± 0.12	1.24
39	1.11 ± 0.03	1.11	1.35 ± 0.01	1.35	1.28 ± 0.02	1.28
52	1.51 ± 0.11	1.48	2.20 ± 0.18	2.16	2.00 ± 0.02	2.01
